# Challenges for the Applications of Human Pluripotent Stem Cell-Derived Liver Organoids

**DOI:** 10.3389/fcell.2021.748576

**Published:** 2021-10-01

**Authors:** Mingyang Chang, Mariia S. Bogacheva, Yan-Ru Lou

**Affiliations:** ^1^Department of Clinical Pharmacy and Drug Administration, School of Pharmacy, Fudan University, Shanghai, China; ^2^Division of Pharmaceutical Biosciences, Faculty of Pharmacy, University of Helsinki, Helsinki, Finland

**Keywords:** human pluripotent stem cells, liver organoid, regenerative medicine, disease modeling, drug development

## Abstract

The current organoid culture systems allow pluripotent and adult stem cells to self-organize to form three-dimensional (3D) structures that provide a faithful recapitulation of the architecture and function of *in vivo* organs. In particular, human pluripotent stem cell-derived liver organoids (PSC-LOs) can be used in regenerative medicine and preclinical applications, such as disease modeling and drug discovery. New bioengineering tools, such as microfluidics, biomaterial scaffolds, and 3D bioprinting, are combined with organoid technologies to increase the efficiency of hepatic differentiation and enhance the functional maturity of human PSC-LOs by precise control of cellular microenvironment. Long-term stabilization of hepatocellular functions of *in vitro* liver organoids requires the combination of hepatic endodermal, endothelial, and mesenchymal cells. To improve the biological function and scalability of human PSC-LOs, bioengineering methods have been used to identify diverse and zonal hepatocyte populations in liver organoids for capturing heterogeneous pathologies. Therefore, constructing engineered liver organoids generated from human PSCs will be an extremely versatile tool in *in vitro* disease models and regenerative medicine in future. In this review, we aim to discuss the recent advances in bioengineering technologies in liver organoid culture systems that provide a timely and necessary study to model disease pathology and support drug discovery *in vitro* and to generate cell therapy products for transplantation.

## Introduction

An organoid is a self-assembled three-dimensional (3D) structure formed by stem/progenitor cells *in vitro*, which can reproduce many structures and functions of an organ (Lou and Leung, 2018). Organoids can be generated from pluripotent stem cells (PSCs), including embryonic stem cells (ESCs) and induced pluripotent stem cells (iPSCs), and tissue-specific adult stem cells. To date, various types of organoids have been generated to mimic tissues of heart, intestine, liver, lung, brain, etc. (Mansour et al., 2018; Miller et al., 2019; Serra et al., 2019; Wang S. et al., 2019; Rossi et al., 2021). Organoid technology represents a significant enhancement of the 3D culture system. The advantage of organoid cultures is that they are combined with bioengineering technology to mimic target organ structure and environment (Yin et al., 2016), and they contain cell types with *in vivo* properties suitable as development and disease models (Collins et al., 2019; Wörsdörfer et al., 2020; Ogoke et al., 2021). Despite the wide applications of organoids, tissue microenvironment, such as cell-cell and cell-matrix interactions, need to support complicated regulatory network, which is important to maintain the homeostasis of an organ. Biological engineering methods have enabled us to guide cell communication and cell behavior to analyze how organs work and to reconstruct the system, which are essential processes in organoid establishment.

Human liver is a structurally and functionally complex organ (Popper and Schaffner, 1957; Weiss et al., 1988), involving distinct cell types and microenvironment and possessing more than 500 functions. The liver consists of endoderm-derived hepatocytes (parenchymal cells) and cholangiocytes and mesoderm-derived sinusoidal endothelial cells, hepatic stellate cells, Kupffer cells, periportal fibroblasts, etc. The liver development requires self-organization of the cells, the process involving biochemical and biophysical cues for morphogenesis and coordinated gene activation/repression, leading to organogenesis (Koepsell et al., 2007; Tam and Loebel, 2007). Therefore, in terms of structural resemblance and functional generalization of the liver organ, liver organoids are superior to cells cultured in two-dimension (2D).

The term liver organoid (LO) refers to 3D multicellular spherical structure made of one or more liver cell types, for example, hepatocyte organoid refers to an organoid formed by hepatocytes. LOs with multiple cell types can better mimic the liver organ while LOs with single cell type are easier to form. LOs can be established from PSCs, fetal, or adult liver cells, and the latter two can be generated directly from human biopsy specimens (Hendriks et al., 2021). Like liver development *in vivo*, PSC-derived liver organoids (PSC-LOs) resemble the structure and functionality of the liver at certain developmental stage depending on the differentiation condition. The crucial problem of PSC-LOs is known to be immature characteristics. Therefore, optimized organoid engineering protocols are continuously being developed to terminally differentiate PSCs into hepatocyte-like organoids (Touboul et al., 2010; Takebe et al., 2017; Pettinato et al., 2019). Mun et al. (2021) claimed for the first time that treatment of human PSC-LOs with short-chain fatty acid mixture of acetate, propionate, and butyrate improved metabolic maturation which may help to accurately assess the CYP3A4-dependent drug toxicity.

LOs are useful for a diverse range of applications, such as studying causes and processes of diseases, gene functions, and cell interaction with tissue environments (van Ineveld et al., 2020; Zhu et al., 2020; Brooks et al., 2021; Thompson and Takebe, 2021; Wang et al., 2021). For *in vitro* applications, LOs have great potential in screening drug hepatotoxicity and modeling liver diseases. One of the causes of high attrition rates is drug-induced liver injury (DILI). In terms of human models of hepatotoxicity, Davidson and Khetani (2020) have developed a scalable culture of human LOs in 384-well plates, which are fully predictive of human DILI and facilitate high-throughput compound screening. For *in vivo* applications, LOs provide hope for cell therapy to treat end-stage liver diseases. However, LOs still face several limitations before they can be used in these applications. Current organoid systems are translationally disadvantaged by variability in self-organization, morphology, and function (Lou and Leung, 2018). Matrigel, used in most cases of organogenesis, still poses the limitation to *in vivo* applications of organoids (Ng et al., 2018; Klotz et al., 2019; Krüger et al., 2020). In this review, we aim to discuss the recent advances in bioengineering technologies in LO culture systems and how bioengineering methods can increase the value of LOs in fundamental research and translational research, such as drug development, precision medicine, and regenerative medicine.

### Types of Liver Organoids

#### Adult Stem Cell-Derived Liver Organoids

Organoid technology has been used to establish hepatic stem cell populations *in vitro* (Schneeberger et al., 2020). A standard adult stem cell-derived LO culture system is a highly potent platform for modeling adult liver from patients’ tissue specimens (Schene et al., 2020). LOs can be established from single EpCAM^+^ cholangiocytes and expanded for several months, while retaining key functional and molecule features after long-term expansion (Huch et al., 2015). It is well-known that adult primary hepatocytes do not replicate *in vitro*, but recent LO studies have allowed them to become highly proliferative, resembling proliferating hepatocytes upon partial hepatectomy or inflammation. Hu et al. (2018) have reported long-term culture of adult hepatocyte-derived organoids that consist of progenitors and differentiated hepatocytes. The researchers confirmed that these hepatocyte organoids were derived from albumin-positive hepatocytes rather than EpCAM^+^ and SOX9^+^ ductal cells. In another study, researchers utilized a regenerative cytokine TNFα to establish long-term expansion of hepatocyte organoids from adult hepatocytes, which mimics inflammation-induced liver regeneration (Peng et al., 2018).

Adult stem cell-derived LOs can be expanded seemingly indefinitely, however, these organoids are derived from a single germ layer, i.e., endoderm, which have limited potential, for example, in modeling complicated liver diseases that involves endoderm- and mesoderm-derived cells. It is tempting to expect that appropriate conditions can facilitate co-culture with mesoderm-derived cells to form liver organoids with significant degree of cellular functionality and architectural complexity.

#### Cancer Derived Liver Organoids

The liver cancer cell line hepatoma G2 (HepG2) and patient-derived tumor xenografts (PDTXs) have long been used as tumor models and have significantly contributed to drug discovery for cancer therapy. 2D immortalized cell lines can easily proliferate *in vitro*, but their gene expression characteristics are highly altered that cannot recapitulate the function of cell types *in vivo*. Generation of PDTXs is labor intensive and time consuming. On the other hand, the generation of patient-derived tumor organoids (PDTOs) is faster. PDTOs have heterogeneous genetic features and can completely simulate tumor characteristics *in vivo*. Thus, PDTOs have enormous potential for modeling human cancers (Kuo and Curtis, 2018; Muthuswamy, 2018) and have been increasingly used in drug development and clinics for personalized drug treatment.

Broutier et al. (2017) have established primary cancer LOs that recapitulate parental tumors, even after long-term expansion *in vitro*. Diverse *in vitro* culture methods, such as bioengineering organoid, allow modeling of tumor heterogeneity or immunity. Cancer LO platform combined with the immune system, angiogenesis, and fibroblasts that retain tumor cell heterogeneity has become a cancer model for cancer microenvironment research and will unleash great potential in evaluating anti-cancer drug efficacy (Papapetrou, 2016; Pauli et al., 2017; Vlachogiannis et al., 2018).

#### Human Pluripotent Stem Cell-Derived Liver Organoids

The hepatic differentiation of human PSCs starts with definitive endoderm (DE) differentiation followed by the formation and expansion of hepatic progenitors and the formation and maturaton of fetal hepatocytes. The generation of PSC-LOs may be performed either partly in 3D, for example, the formation of DE cells (Akbari et al., 2019), hepatic progenitors (Wang S. et al., 2019), or hepatocyte-like cells (Sgodda et al., 2017) in 2D condition followed by the transfer of obtained cells into 3D condition for the maturation, or completely in 3D from the beginning of the PSC stage (Guan et al., 2017). Hepatic endoderm cells can aggregate into 3D structures in certain conditions, particularly as a result of the activation of FGF and BMP signaling pathways (Takebe et al., 2013).

Human PSCs-LOs can contain single or multiple cell types, such as hepatocytes, cholangiocytes, and other non-parenchymal cells, which self-organize to form the structural units present in the liver. As a result, they are closely mimicking the complex structure and functionality of the liver.

Human PSC-LOs rely on the self-organizing ability of stem cells and progenitors to form organized structures for modeling developmental processes of liver organogenesis. To better control the differentiation processes and decrease heterogeneity, organoids should be designed to generate a specific liver region according to liver zonation. *In vitro* models mimicking liver zonation have been established by HepaRG cells and hepatocytes from neonatal rats (Ahn et al., 2019; Janani and Mandal, 2021).

### Liver Diseases

Liver disease is one of the leading causes of death worldwide. According to the National Center for Health Statistics in the US, the morbidity of adults with diagnosed liver disease is 1.8% and is expected to continue in the next decades (National Center For Health Statistics, 2021). Common causes of chronic liver disease and cirrhosis are viruses, genetics, autoimmune disease, excessive alcohol use, and obesity. Acute liver failure, also known as fulminant hepatic failure, is caused by drugs or toxic chemicals. These are severe damage factors to the liver, and at a certain point in the progression of liver disease, the injury can become irreversible and lead to end-stage liver disease, liver failure, liver cancer, or death. Liver transplant is the only effective treatment for end-stage liver diseases. The number of patients on waiting lists well exceeds organ donation rates and therefore people have high expectation for human PSC-derived liver cells as an alternative treatment. Earlier studies have demonstrated the feasibility of human PSC-derived liver cells as cell therapy (Takebe et al., 2017). Additionally, human PSC-LOs have potential as *in vitro* models in drug discovery and development.

## Challenges for the Applications of Human Pluripotent Stem Cell-Derived Liver Organoids

### Regenerative Medicine

As human PSC-derived cells represent a substitute to cadaver and organ transplantation, human PSC-LOs provides an avenue toward cell therapy. By using improved organoid technology and transplantation techniques, transplanted human PSC-LOs could potentially integrate and grow *in vivo* into functional liver tissue to replace injured hepatocytes and non-parenchymal cells that are caused by liver diseases. Nevertheless, before human PSC-LOs are suitable for regenerative treatment, many issues must first be solved.

#### Xenogeniciy of Biomatrices

In the process of organoid formation, one aspect is the induction of stem cell differentiation that requires a large number of growth factors or small molecules that are tissue-specific and can change signaling pathways for cell survival, migration, and proliferation. The other aspect is the formation of 3D tissue-like structures that rely on 3D cell culture environments. When building a 3D cell culture environment, it is necessary to consider extracellular cues influenced by the scaffold, the composition of extracellular matrix (ECM) proteins, and the stiffness of the matrix. Matrigel-based matrix is often used for the generation and culture of various PSC-derived and tissue-specific stem cell-derived organoids including liver (Mun et al., 2020), brain (Lancaster et al., 2013), kidney (Takasato et al., 2015), and intestine (Sugimoto and Sato, 2017). Matrigel provides scaffolding and signaling by forming basement membranes to support cell attachment and functionality, including organoid formation (Xu and Zeger, 2001). Ouchi et al. (2019) embedded human PSC-derived foregut spheroids into Matrigel to create LOs. The resultant LOs consist of hepatocytes, hepatic stellate cells, and Kupffer cells with specific polarity and more mature characteristics in comparison with fetal human hepatocytes. A simultaneous induction of DE and mesoderm cells on Matrigel is another approach resulting in the formation of LOs containing two cell types—hepatocytes and biliary cells (Wu et al., 2019). Although Matrigel has great potential to support the generation of LOs, it is a basement membrane extracted from the Engelbreth-Holm-Swarm mouse sarcoma (Orkin et al., 1977), which severely limits organoid application in clinical practice.

##### Xeno-free biomatrices

Efforts have recently been made in developing clinically acceptable matrices or biomaterials for LOs (Table 1). The first class is animal-derived matrix, such as collagen type I, decellularized matrix, and hyaluronic acid. Collagen type I is the key component of the liver ECM that affects cell growth, viability, differentiation, and overall tissue organization. As xenogeneic collagen and hyaluronic acid are clinically acceptable, they can be used as a scaffold for the generation of LOs. Mixing of iPSC-derived hepatocyte-like cells with collagen solution followed by heating for the induction of vitellogenesis led to the generation of a 3D structure with an *in vivo*-like architecture (Gieseck et al., 2014). The transfer of clumps of 2D iPSC-derived hepatocyte-like cells into 3D collagen-based scaffold doubled the percentage of glycogen-synthesizing cells, increased the expression of mature hepatic genes, decreased the expression of fetal liver markers AFP and CYP3A7, and promoted the establishment of cell polarity. The long-term stable functionality of cells in 3D makes this model a promising tool for toxicity assessment. Moreover, this model is suitable for high-throughput screening studies (Gieseck et al., 2014).

**TABLE 1:** 3D cell culture systems for human LOs.

3D cell culture system	Xenogenicity	Type	Cell types in LOs	Aim	Limitation	References
Matrigel	Mouse-derived	Natural	Human iPSC-derived hepatocyte-, stellate-, and Kupffer-like cells	Fibrosis model	Clinically unacceptable	Ouchi et al., 2019
Matrigel	Mouse-derived	Natural	Human iPSC-derived hepatocytes and biliary cells	Hepatobiliary organogenesis	Clinically unacceptable	Wu et al., 2019
Collagen hydrogel	Animal-derived	Natural	Human iPSC-derived hepatocyte-like cells	Hepatic maturation		Gieseck et al., 2014
Decellularized matrix	Porcine intestine-derived	Natural	Human liver duct cells or fetal hepatocytes	Clinical applications	Batch-to-batch variation	Giobbe et al., 2019
Alginate capsules	Plant-derived	Natural	PSC-derived hepatocytes and stromal cells	Functional engraftment		Song et al., 2015
Nanofibrillar cellulose hydrogel	Plant-derived	Natural	Human adult liver cells	Clinical applications	Non-biodegradable	Krüger et al., 2020
Colloidal crystal scaffolds with collagen type I	Partially animal-derived	Synthetic/natural	Human iPSC-derived hepatic progenitors	Fully defined matrix for clinical applications		Ng et al., 2018
Poly isocyanopeptides and laminin-111	Not animal-derived	Synthetic/natural	Human adult liver cells	Clinical applications		Ye et al., 2020
Poly (ethylene glycol) (PEG) hydrogels	Not animal-derived	Synthetic	Human adult liver cells	Chemically defined for clinical applications		Sorrentino et al., 2020
Matrix-free suspension	None	None	Human ESC-derived hepatocyte-like cells	Hepatic maturation		Ogawa et al., 2013
Matrix-free suspension	None	None	Human ESC- derived hepatocyte-like cells	Large-scale expansion		Sgodda et al., 2017

Owing to its complex *in vivo*-like properties, decellularized matrix is used more often to support cell expansion and differentiation than any existing matrix components (Giobbe et al., 2019). After decades of research, especially in recent years, the need for complex forms of ECM has been clarified (Manou et al., 2019). So far, better results have been obtained using matrix extracts prepared by decellularizing cartilage and myocardium tissues (Schwarz et al., 2012; Oberwallner et al., 2014). Ott et al. (2008) have previously developed a technology for organ decellularization that uses a detergent for cell removal from the heart. Decellularized scaffold retains tissue-specific 3D architecture and vascular network. The decellularized scaffolds have a wide variety of applications in reconstruction of liver tissue or organ (Uygun et al., 2010; Baptista et al., 2011; Minami et al., 2019). The low immunogenicity makes decellularized matrix clinically applicable.

Rat decellularized liver scaffold has been used for the formation of human iPSC-derived hepatocyte grafts after differentiation of the iPSCs into hepatocytes in 2D culture. Recellularization of the scaffold with iPSC-derived hepatocytes led to the expression of CYP3A4 enzyme and secretion of albumin but in a lower amount than 2D iPSC-derived hepatocytes (Minami et al., 2019). Although the maturity of the recellularized iPSCs-derived grafts was not high enough and needs more study for the improvement of the protocol, the important outcome of this research is the demonstration of the suitability of the xenogeneic decellularized scaffold for human liver engineering. Effective engraftment and high induction of hepatocyte and cholangiocyte markers were reached in hepatic stem cells cultured and differentiated in the decellularized liver matrix (Wang et al., 2011), showing the potential of decellularized scaffold for liver bioengineering. Decellularized ECM promotes higher CYP enzyme activity in iPSC-derived hepatocytes compared with cells cultured in synthetic poly-l-lactic acid scaffold covered with collagen (Wang et al., 2016).

Decellularized ECM in combination with a linear polysaccharide hyaluronic acid has been shown to support the tight junction formation and possessed a lower immune response compared to other natural hydrogels (Deegan et al., 2016). Immunotolerance is one of the advantages of decellularized ECM, allowing its use in transplantation. Hyaluronic acid is an important component of the natural ECM. It is easy to modify hyaluronic acid *in vitro* to adjust its stiffness and other physical parameters according to its intended application. Particularly, hepatocytes grown in the hyaluronic acid hydrogel in 3D condition possessed high viability and growth rate (Burdick and Prestwich, 2011).

It remains challenging to obtain scalable and well-controlled organoids due to the complex composition and batch-to-batch variability of human or animal-derived matrices. Therefore, some plant-based biomaterials have been developed. Alginate, a marine algae water-soluble polysaccharide copolymer, has been successfully used for 3D cell culture. Alginate hydrogels are biocompatible, non-immunogenic, and hydrophilic (Fedorovich et al., 2007). Song et al. (2015) differentiated iPSCs into hepatocyte-like cells in a 2D environment, then co-aggregated them with stromal cells in a matrix-free environment, and then encapsulated the generated 3D organoids in alginate capsules and transplanted them into mice. The transplanted organoids demonstrated high albumin and α-antitrypsin secretion compared with primary human hepatocytes. Nanofibrillar cellulose hydrogel (also called cellulose nanofibril hydrogel, CNF) is a plant-origin xeno-free, non-toxic, and biocompatible hydrogel that has been successfully implemented for the 3D culture of human PSCs (Lou et al., 2014). Recently, CNF hydrogel was used to generate human adult liver-derived LOs (Krüger et al., 2020). The CNF was used for LO expansion due to its mechanical properties. It also provides a supportive environment to induce LOs to functional hepatocyte-like cells. Thus, the CNF hydrogel presents a viable alternative to Matrigel for clinical use.

Unlike natural biomaterials mentioned above, synthetic scaffolds possess adjustable features and users can modify stiffness, swelling rate, etc., as well as modify the scaffold with functional groups. A well-defined composition of synthetic scaffolds provides reproducible results. Ng et al. (2018) used inverted colloidal crystal scaffolds with type I collagen coating for the maturation of iPSC-derived hepatic progenitors. They have proved that the morphological and transcriptomic features of those hepatic progenitors were reached and overrode the liver characteristics level of the spheroids cultured on Matrigel. Pore size was claimed as an important factor that allowed cells to form spheroids. They demonstrated that the most optimal pore diameter for this purpose is 140 μm. The appropriate ECM-mimicking coating was shown necessary for the cell attachment when the scaffold is formed from the biologically inert material.

Ye et al. (2020) have recently provided a novel hydrogel based on polyisocyanopeptides and recombinant human laminin-111, which is promising for human adult liver-derived LO culture over at least 14 passages. Moreover, they have recently shown that the elasticity of tissue ECM is critical to many cell types. It is believed that ECM elasticity can help cells develop and function (Sorrentino et al., 2020). By adjusting the composition and elasticity of an artificial matrix, we can simulate the properties (e.g., elasticity, extensibility) of the target tissue through the composition of the ECM. This result is of great significance to the maintenance of cell viability and function, the establishment of disease models, and tissue repair and regeneration.

In summary, matrix-based culture condition needs to be further improved to exclude immunogenic and clinically unacceptable matrix and biomaterials, so as to be used for regenerative medicine and transplantation.

##### Matrix-free systems

Matrix-free or biomaterial-free 3D culture does not have any problems related to xenogeneic matrix and thus can be easily adapted for clinical applications. Ogawa et al. (2013) demonstrated that the maturation of hepatocyte-like cells can be increased through the transferring of human ESC-derived hepatocyte-like cells to the matrix-free suspension environment. Another study also formed LOs by aggregating PSC-derived hepatocyte-like cells (Sgodda et al., 2017). In this case, hepatocytes aggregated into organoids within 12 h. This protocol allows better control of the differentiation in 2D, and subsequent transfer into 3D culture led to the continuation of the differentiation that was determined by the change of the expression pattern of maturation genes. The authors also demonstrated the importance of size control of the LOs, indicating that the increase in the size is associated with the decrease in the hepatic functions.

In conclusion, for applications in regenerative medicine, the major xenogenic materials used in PSC-LO generation are biomaterials, such as Matrigel, which should be replaced with clinically acceptable biomaterial or removed when using suspension culture system.

#### Genetic and Epigenetic Instability

The iPSC technology offers full of promise for cell therapy (Takahashi et al., 2007; Yu et al., 2007; Mandai et al., 2017). The differentiation of iPSCs can generate tissue-specific cells for transplantation, and can also apply to *in vitro* studies and drug development (Grskovic et al., 2011; Inoue and Yamanaka, 2011; Plummer et al., 2019). One of the most important safety issues particularly related to the *in vivo* applications of PSC-derived cells are genetic and epigenetic instability, resulting in variations. These variations can occur at distinct levels, mainly during the generation and maintenance of iPSCs and the differentiation and expansion of iPSC-derived cells. The genetic and epigenetic variations or unstable chromosomes may change the characteristic of iPSCs and affect differentiation capacity (Nishizawa et al., 2016), yet the instability has not been fully elucidated.

##### Variations introduced into induced pluripotent stem cells

The variations may originate from the heterogeneous genetics of source cell population (Huang, 2009). Moreover, if some specific variations in source cells effectively promote reprogramming, these variations will be amplified in the derived iPSCs (Cahan and Daley, 2013; Kilpinen et al., 2017). Burrows et al. (2016) compared human iPSC lines from two somatic cell types of four donors in terms of DNA methylation and gene expression and found that genetic variation between donors, but not between cell types, is the main cause for the differences between iPSC lines. By studying 711 human iPSC lines, Kilpinen et al. (2017) have found that 5–46% of variations were originated from differences between individuals (Cahan and Daley, 2013).

In addition, reprogramming protocols may introduce new variations by increasing mutations. Integrative vectors, such as lentivirus and retrovirus, can randomly integrate into the genome of iPSCs to interrupt endogenous gene expression and reactivate transgenes to lead to tumorigenesis, whereas non-integrating vectors, such as Sendai virus, adenovirus, and adeno-associated virus, induce transient expression of transcription factors. A whole exome sequencing study identified mutations in human iPSCs, of which 75% occurred during reprogramming process (Ji et al., 2012). Although the exact reason for mutagenesis was not identified in the study, retrovirus used in reprogramming could be one of the drivers. On the contrary, a later study compared three reprogramming methods, namely retrovirus, Sendai virus, and synthetic mRNA and found that the number of genetic variants identified by whole genome sequencing was moderate and did not differ between reprogramming methods (Bhutani et al., 2016). The authors concluded that reprogramming was unlikely to make human iPSCs unacceptable in cell therapy. Nonetheless, non-integrative vectors are recommended in clinical applications.

Like ESCs, iPSCs maintenance may introduce genetic or epigenetic alterations into cells. During iPSC culture, there were genetic variations among different passages or among different populations (Mayshar et al., 2010; Amps et al., 2011). These studies found that the level of overexpressed genes increased with the increased passage number and in some case, normal iPSCs at lower passage number exhibited gains in some chromosomes. Genetic and epigenetic variations preexisting in source cells or being introduced during reprogramming are subjected to selection in prolonged culture (Liang and Zhang, 2013). A high-resolution single nucleotide polymorphism genotyping study has found that duplications of oncogenes tend to accumulate during prolonged passaging of human iPSCs (Laurent et al., 2011). An unbiased study using clonal culture has identified oxidative stress as the trigger for mutation accumulation during passaging and showed that the mutation rate in human iPSCs was lower than that in human intestinal and liver stem cells (Kuijk et al., 2020).

##### Variations introduced into induced pluripotent stem cells-derived cells

Genetic and epigenetic instability introduced during the differentiation of human PSCs has not been well investigated. A study using human parthenogenetic stem cells has accessed instability during a serial round of differentiation and reprogramming, i.e., differentiating parthenogenetic stem cells into parthenogenetic mesenchymal stem cells via the formation of embryoid body and reprogramming parthenogenetic mesenchymal stem cells into iPSCs using retrovirus-mediated delivery of OCT4, SOX2, KLF4, and c-Myc (Vassena et al., 2012). When comparing the first-round parthenogenetic mesenchymal stem cells with the second-round parthenogenetic mesenchymal stem cells by microarray analysis, the authors found more than 3,000 differentially expressed genes and concluded that these differences were introduced during reprogramming, though no evidence was provided to show the differences appeared during reprogramming, not during differentiation. Nonetheless, this study draws our attention that genetic and epigenetic instability should be examined throughout the process of reprogramming and differentiation to ensure safe cell therapy in regenerative medicine.

##### Impact of genomic instability and coping strategies

Many researchers have proven that different lines of iPSCs have diverse differentiation and developmental capability (Polo et al., 2010; Tsuji et al., 2010; Boulting et al., 2011; Kim et al., 2011; Liang and Zhang, 2013). This diversity is caused by cell of origin or genetic and epigenetic variations in iPSCs. Some of these variations may result in potential abnormalities in iPSC differentiation and induction, and thereby causing phenotypic changes and functional deficiencies, which pose risks in their *in vivo* applications as well as problems in disease modeling and drug development (Mekhoubad et al., 2012).

There are some coping strategies to reduce the iPSC variability, such as reducing the causes of variations in source cells and optimizing reprogramming methods and culture conditions. First, the preexisting mutations in source cells vary with the cell of origin. Ultraviolet-induced somatic mutations in skin fibroblasts were found in fibroblast-derived human iPSCs (D’Antonio et al., 2018). For this reason, hematopoietic stem cells show advantages over skin fibroblasts as a safe cell source (Wang K. et al., 2019). Second, reprogramming method should be carefully selected regarding integration, genomic instability, and tumorigenesis issues. Third, it is necessary to acquire enough cell numbers of iPSCs for differentiation studies and applications. In light of this purpose, genetic and epigenetic variations in iPSCs should be detected and monitored throughout passages (Martins-Taylor et al., 2011). Assou et al. (2020) have developed a test which can potentially detect more than 90% of human PSC recurrent genetic abnormalities from long-term culture and can be used to routinely screen genomic integrity in human PSCs. The test was established based on a large dataset of reported genetic and epigenetic abnormalities and uses droplet digital PCR technology which greatly simplifies the regular and systematic hPSC monitoring. In addition, it would be ideal to set up a bank of human iPSC and ESC lines, in which each line is maintained as a homogeneous and genetically stable population. To set up a transgene-free human iPSC bank enabling high-throughput generation and rapid expansion to meet industrial and clinical demands, Valamehr et al. (2014) have established a platform using small molecule pathway inhibitors in feeder-free culture condition. Further studies should focus on clarifying the genetic and epigenomic stability during the *in vitro* differentiation process, such as using chemically defined reagents to reduce variability and optimal oxygen condition, for the safe application of iPSCs-derived cells in regenerative medicine.

#### Genome Editing

Genome editing can be used to add, remove, or edit DNA of cellular genome to alter the characteristics of a cell or an organism, and it has the potential to both improve our understanding of human genetics and cure genetic diseases (Hou et al., 2013; Ran et al., 2015). The most used tool of genome editing is the RNA-guided CRISPR-Cas9 nuclease system that has emerged in recent years representing a system that is easy to design, highly specific, efficient, and suitable for high-throughput and multiplexed gene editing.

Since the establishment of human iPSCs, researchers had hoped to use patient’s own iPSC-derived cells in regenerative medicine. However, there are four problems in autologous iPSC-based cell therapy: (1) the process of establishing autologous cell therapy is complex, time-consuming, and costly. This expensive treatment is difficult to promote and attract pharmaceutical companies to develop. (2) Autologous cell therapy is difficult to standardize. Quality control faces great challenges. (3) Autologous cell therapy cannot treat acute diseases, such as acute liver failure. (4) In the process of differentiation, the immunogenicity of autologous iPSCs will change, and the generated cells may not be immunotolerant as reported earlier (Zhao et al., 2015). On the contrary, allogeneic cells are easier to be standardized as a treatment, and can be mass produced into off-the-shelf products. However, immune rejection in allogeneic cell transplantation remains to be overcome. HLA matching iPSC lines have been established for allogeneic cell transplantation (Okita et al., 2011; Taylor et al., 2012). In recent years, scientists have established several methods to overcome the immune rejection of allogeneic PSCs. For example, CRISPR-Cas9-mediated destruction of HLA gene enhanced the immune compatibility of iPSCs (Hong et al., 2017; Xu et al., 2019; Lee et al., 2020). These studies about allogeneic human PSC-derived cells without immune responses as cell therapy open a new avenue toward regenerative medicine.

Allogeneic PSC-LO-induced immune rejection is primarily mediated by T cells. Strategies to regulate T cell costimulatory and inhibitory pathways can prevent immune rejection. A study using monoclonal antibodies to block T cell costimulation has shown immune tolerance of human ESC-derived pancreatic endoderm cells in mice (Szot et al., 2015). However, this strategy has a potential risk for cancer and infection.

Despite the obvious advantages that gene-edited iPSC-based cell therapy has, challenges like off-target, mutagenesis, tumorigenesis, and ethical debate may still exist. One of the major causes of the off-target effects of the CRISPR system is the continusly expressed Cas9 proteins in cells (Fu et al., 2013). Random integration resulted from off-target effects can potentially develop insertional mutagenesis and subsequent tumorigenesis of transplanted cells. Thus, in order to reduce the off-target effects caused by Cas9 overexpression, strategies, such as decreasing the amount of undesirable DNA cleavage or suppressing the activity of Cas9 protein, have been suggested (Nuñez et al., 2016). The use of a high-fidelity Cas9 variant is another strategy to reduce off-target events (Kleinstiver et al., 2016). We recently developed a Cas9 mRNA-based CRISPR genome editing method to efficiently edit human PSCs (Leung et al., 2020). We show that Cas9 existed in cells in a short period time. Like Cas9 ribonucleoprotein, our method can produce cells without foreign gene integration and thus can be easily translated into clinical applications. Thus, gene-edited PSCs together with organoid technology provide more opportunities in regenerative medicine.

#### Large-Scale Expansion for Transplantation

For future therapeutic applications of human PSC-derived liver cells, the number of cells required for each transplantation will be quite large, about one tenth of the liver mass which is approximately 10^9^ cells (Sharma et al., 2011). An earlier strategy was to expand human PSCs, and more recently researchers have found some ways to expand differentiating PSCs at certain developmental stages, such as endodermal cells using defined growth factors (Raju et al., 2017) and hepatoblasts by combining growth factors and small molecules (Zhang et al., 2015). These expanding hepatoblasts maintain phenotypes during long-term culture and can differentiate into mature hepatocytes and bile duct cells. The aim was to generate a source for cell therapy of liver diseases.

Organoid technology has recently been used to expand various types of cells *in vitro*. Wang S. et al. (2019) developed a differentiation cocktail medium for the differentiation of human ESCs into LOs with high expansion ability. Hepatic progenitors derived in 2D culture conditions were embedded in Matrigel and treated with a unique combination of B27, EGF, Wnt-3A, Forskolin, N2, Nicotinamide, N-acetylcysteine, R-spondin, Gastrin, and A83-01 compound. This approach allowed long-term subculture of the resultant organoids (up to 20 passages) that remained stable after freezing-thawing procedures and mass-scale production of cells which is necessary for industrial applications. Another study showed that self-aggregated PSC spheroids formed in agarose microplates can differentiate in suspension into 3D liver tissue whose phenotype was stable for over 1 year without detectable tumorigenic activity after transplantation (Rashidi et al., 2018). The ability of the long-term culture of human PSC-LOs that remain their functional activity is one of the main advantages over primary human hepatocyte-derived organoids due to the limited supply of primary human liver tissue from healthy or diseased liver donors. Other researchers have generated LOs from human PSC-derived endoderm cells in 2 weeks and expanded them in Matrigel for more than 16 months without loss of differentiation capacity (Akbari et al., 2019). LOs spontaneously formed from 2D culture of differentiating human PSCs were expanded in Matrigel for 1 year while maintaining their karyotype and phenotype (Mun et al., 2020).

In summary, great efforts have been made to expand differentiating cells for large-scale production. However, the use of Matrigel must be replaced by a xeno-free matrix or biomaterial to meet clinical requirements. For regenerative medicine, expansion in suspension culture (Rashidi et al., 2018) seems superior than Matrigel culture.

#### Transplantation and *in vivo* Engraftment

Cell transplantation has potential for end-stage liver diseases (Tsuchida et al., 2020). Human PSC-LOs as a new source for cell transplantation is innovative but still at the preclinical stage. During the process of transplantation, the homing and survival of transplanted cells are major factors determining the success of transplantation. At present, there are no standard protocols defining the differentiation status of transplanted cells (fetal stage or mature stage), cell formulation (single cell suspension, organoid suspension, or biomaterial-based construct), transplantation site (systematic, orthotopic, or ectopic), and delivery technique (injection, infusion, or implantation).

The most commonly used method to deliver liver cells or LOs is injection, which involves intraportal injection, intravenous infusion, intramuscular injection, or intrasplenic injection. Intrasplenic or portal venous infusion can hardly control engraftment efficiency and avoid ectopic engraftment because transplanted cells first enter the blood circulation before reaching the liver. A recent preclinical study in pigs has reported that the ligation of the patent ductus venosus before portal venous infusion can inhibit extrahepatic translocation (Tsuchida et al., 2020). In intrasplenic transplantation, more than 70% transplanted cells are rapidly removed by resident immune cells (Gupta et al., 1999). An earlier study has found that the disruption of sinusoidal endothelium can facilitate the integration of transplanted cells into the liver (Gupta et al., 1999). Rashidi et al. (2018) transplanted human PSC-LOs in two ways, intraperitoneal injection and subcutaneous implantation with the aid of a polycaprolactone scaffold. Both methods improved the liver functions of diseased recipient mice, but the authors did not compare these two methods, only suggesting that ectopic implantation is less invasive. Tsuchida et al. (2019) recently reported that human iPSC-LO transplantation via the portal vein had good retention of organoids in the liver, whereas single cells of LOs translocated to the lung. Some studies have found that immature cells can further become mature once transplanted *in vivo* (Takebe et al., 2013), but there is still no solid evidence on the differentiation status of transplanted cells for the best functional engraftment while avoiding tumorigenesis of immature cells.

As an alternative to liver organ transplantation for liver failure, liver cells or LOs could directly implanted to the liver orthotopically. Nagamoto et al. (2016) have implanted a cell sheet composed of human iPSC-derived hepatocytes onto the liver surface of recipient mice with acute liver injury induced by CCl_4_. The orthotopic cell sheet transplantation exhibited better cell retention than intrasplenic injection, and thus had increased engraftment rate and efficacy. The authors also pointed out that a large number of cells can be transplanted using the cell sheet technology. To deliver LOs by the cell sheet technology, LOs need to be dissociated into single cells and then cultured in 2D to form a cell sheet. However, dissociation of LOs will disrupt tissue structure and may be incompatible with LO technology.

Despite many innovative transplantation methods, the low engraftment efficiency might be due to poor integration of human iPSC-derived cells to the local environment or the lack of vasculature in LOs. To evaluation transplantation and engraftment efficiency, we need non-invasive *in vivo* cell tracking methods (see the section below) (Tsuchida et al., 2020).

#### *In vivo* Cell Tracking

Real-time monitor of transplanted cells or organoids *in vivo* is necessary to study the fate of transplanted cells and evaluate engraftment during preclinical studies (Watson et al., 2014; Jung et al., 2018; Hsia et al., 2021). Ideally a reporter with highly sensitive 3D tomography is needed to non-invasively measure the engraftment of transplanted hepatic cells. Non-invasive measurement allows short, long-term, and repeated monitoring transplanted cells *in vivo*. The signal of an ideal reporter needs to last long and have resolution in certain depth to reach internal organs and at micrometer scale to localize cells. A study showed that human sodium iodide symporter (hNIS) can serve as such a reporter and can utilize radiotracers already available for clinical use to enable positron emission tomography or single photon emission computed tomography (Ashmore-Harris et al., 2019). In this study, human iPSC-derived immature hepatocytes were transduced with lentivirus containing dual-mode radionuclide-fluorescence hNIS-mGFP reporter gene followed by further differentiation *in vitro* and transplantation *in vivo*. The signal of hNIS-mGFP reporter was stable and indicating the transplanted cells precisely homing to the liver, but the signal became undetectable 1 week later due to cell death in the *in vivo* environment.

Another *in vivo* tracing technology is Raman spectroscopy-based modality that can mine data, such as proteomic and chemogenomic data. Coherent Raman scattering (CRS) microscopy is a high-speed vibrational imaging system that can visualize the chemical content of a living specimen. The major technique in CRS is coherent anti-Stokes Raman scattering (CARS) (Reintjes et al., 1982). With technical advances on hyperspectral CARS microscopy, researchers have provided many innovation studies on organoids. Pope et al. (2021) have investigated a complex multicellular system of LOs by using correlative two-photon fluorescence and hyperspectral CARS microscopy. Their most interesting finding is that organoids could be maintained alive under hyperspectral CARS measurements. In addition, they have established a method for label-free identification of chemically distinct subpopulations, which can be used to analyze and validate the quality of clinical cell transplantation. These studies emphasize the future of quantitative hyperspectral CARS microscopy as an empowering technology in regenerative medicine, showing the way to novel possibilities for non-invasive disease diagnosis.

In conclusion, to develop human PSC-LOs as a therapeutic product, safety, efficacy, and controllable quality must be fulfilled. Safety consideration includes genetic and epigenetic stability, non-xenogenicity, immune tolerance, and non-tumorigenesis. Efficacy is influenced by PSC-LO quality and delivery method. It still remains unclear on the effect of human PSC-LOs transplanted into the liver. Non-invasive *in vivo* cell tracking methods can help evaluate engraftment efficiency and cell fate.

### Disease Modeling and Drug Development

Organoid technology is a powerful tool for the study of human diseases. LOs generated from patient-derived or genome-edited PSCs have the ability to model liver diseases, to screen drug candidates, and to test their toxicity. Liver is the main organ for drug metabolism and transport. It is also the main target organ of drug toxicity. An adverse drug reaction Peng et al. (2018) is an undesirable side effect of a drug and is classified as dose-dependent or idiosyncratic drug-induced damage. Historically, toxicology research has relied on animal models to characterize the toxicity of new compounds. However, animal models are not perfect systems for human toxicity, and it can only predict 50% of DILI in humans. Thus, human toxicity prediction models are urgently needed. Primary adult human hepatocytes are the “gold standard” for evaluating drug metabolism and toxicity. However, due to their limited availability and proliferation capacity, alternative *in vitro* models are needed. Human PSCs represents an unlimited cell source for all the cell types in human body and are expected to generate *in vitro* models for drug discovery and development (Lin et al., 2021). The applications of human PSC-derived liver cells include liver disease modeling, drug metabolism, and hepatotoxicity study. As mentioned in see section “Introduction,” PSC-LOs show superior structural and functional advantages over 2D cultured PSC-derived liver cells. Guan et al. (2017) estabilished a disease model of JAG1 mutations based on human iPSC-LOs with bile duct–like structures. They adopted iPSC-based organoid system and genome editing technology to show the effect of mutations on human genetic diseases. For disease modeling and drug development, a number of challenges related to human PSC-LOs remain to be overcome. The major challenge of human PSC-LOs is immaturity of hepatocytes derived from current differentiation protocols (Lynch et al., 2019). Heterogeneity is another problem related to organoid technology in general (Lou and Leung, 2018), which reduces reproducibility of drug screening and testing. Building PSC-LOs with multiple cell types to mimic liver tissue complexity is another goal, which is particularly necessary when modeling liver diseases involving several cell types or studying DILI as hepatocyte damage is caused not only by direct toxic effect of drugs but also by indirect effect of non-parenchymal cell response.

#### Immaturity

Mature PSC-LO models should show basic liver functions and tissue environmental characteristics. The studies focusing on liver development have contributed to establishing PSC induction methods and mimicking the environment for fetal liver cell development and growth. Liver matures during perinatal period. However, due to the limited knowledge on perinatal human liver development, the immaturity of human PSC-LOs still remains an unsolved problem. This problem hinders their applications in disease modeling and drug development. Currently, the differentiation of hepatic progenitors is mediated by HGF, oncostatin M (Prodanov et al., 2016), and dexamethasone, but the resultant hepatocytes exhibit fetal liver features (Baxter et al., 2015), such as the expression of fetal marker alpha-fetoprotein (Lucendo-Villarin et al., 2020) and lower activity of CYP3A4 than primary hepatocytes (Lee et al., 2021).

In recent years, researchers have improved the hepatic maturation of human PSC-LOs by using different factors and small molecules. According to the changes in liver microenvironment at postnatal stage, researchers have found that microbial short-chain fatty acids could improve the metabolic functions of human PSC-LOs (Mun et al., 2021). Wu et al. (2019) have firstly established functional hepatobiliary organoids from human iPSCs. 25% of mTeSRTM culture medium was replaced with RPMI-1640/B27 minus insulin at differentiation stages to induce hepatic and biliary co-differentiation, then 10% cholesterol^+^ MIX was added to promote maturation. Cholesterol^+^ MIX is a preparation from Chinese medicine products, mainly comprised of cholesterol and other small molecules.

In addition, the role of non-parenchymal cells in liver maturation has also drawn attention. Asai et al. (2017) observed that iPSC-derived hepatic endoderm cells directly interacted with human umbilical vein endothelial cells (HUVECs) and mesenchymal stem cells during liver organoid morphogenesis. At the same time, HUVECs might also exert paracrine effects, such as secreting HGF, to promote hepatocyte differentiation. Another study shows that non-parenchymal cells, namely mesenchymal stromal cells and endothelial cells, improved the hepatic maturation of human PSC-LOs by decreasing TGF-β and Wnt signaling pathways (Goulart et al., 2019).

ECM plays an important role in cell differentiation and organogenesis and may improve hepatic maturation of PSC-LOs. Zahmatkesh et al. (2021) efficiently improved hepatic maturation of LOs by mixing microparticles made of decellularized liver matrix with human PSC-derived endoderm cells, mesenchymal stromal cells, and HUVECs.

In conclusion, researchers in the past few years have made efforts to improve hepatic maturation of human PSC-LOs by applying small molecules found in the liver microenvironment during postnatal stage, incorporating non-parenchymal cells, and utilizing ECM components or bioengineering techniques (Table 2). The measurements indicating liver maturity vary a lot among different studies, from synthetic functions to metabolic functions. In some studies, the measurements were not compared with the primary adult human hepatocytes, and thus it is uncertain how mature these LOs are and whether they can be a valid model in disease modeling and drug development. The liver mature features should be selectively measured according to the intended applications of human PSC-LOs. We believe that with the better understanding of *in vivo* liver maturation, we will be able to generate PSC-LOs mature enough for drug screening and toxicity testing.

**TABLE 2:** Strategies for improving maturity of human PSC-LOs.

Strategies	Cell types	Key methods	Representative maturation measures	Model	Comparison	References
Soluble factors	iPSC-derived hepatocytes	Acetate, propionate, and butyrate combination in hepatic medium and differentiation medium	Increased CYP3A4 activity and ALB secretion	Drug-induced liver injury	iPSC-LOs were less sensitive than PHHs to troglitazone-induced toxicity, but HepG2 was not sensitive.	Mun et al., 2021
	iPSC-derived hepatocyte- and cholangiocyte-like cells	25% mTeSR in endoderm medium and 10% cholesterol+ MIX (Chinese medicine) to maturation medium	Bile duct structure and production and transport of bile acids	Hepatobiliary organogenesis	The maturity of iPSC-LOs was between fetal liver and adult liver.	Wu et al., 2019
Co-induction	PSC-derived hepatocytes and cholangiocytes	Hepatic endoderm spheroids were differentiated into hepatoblast spheroids, which were dissociated and seeded in an ultra-low attachment plate to form LOs.	Functional bile canaliculi system	NASH model	Free fatty acid-treated PSC-LOs showed similar gene expression signatures to NASH patients.	Ramli et al., 2020
	Healthy and Wolman diseased PSC-derived hepatocytes-, stellate-, and Kupffer-like cells	PSC spheroids embedded in Matrigel were stepwise differentiated into LOs.	LOs can be induced by free fatty acid to have inflammatory and fibrotic responses. All cell types in LOs are functional.	NASH/Fibrosis model; Wolman LO model	Wolman LOs exhibited more aggressive fibrosis phenotypes than Wolman disease patients.	Ouchi et al., 2019
Co-culture	iPSC-derived hepatocytes, HUVECs, and MSCs	iPSC-derived hepatic endoderm, HUVECs, and MSCs self-organized into 3D liver buds (LBs).	Vascularized and functional human liver	Regenerative medicine	iPSC-LBs produced higher levels of albumin than human adult hepatocytes *in vivo*.	Takebe et al., 2013
	iPSC-derived hepatocyte-like cells, HUVECs, and MSCs	Hepatic-specified endoderm co-cultured with HUVECs and MSCs without cell-cell contact.	After no cell-cell contact co-culture, hepatocyte-like cells had polarity and bile acid transport structure.		Some genes were not upregulated when compared with primary hepatocytes.	Asai et al., 2017
	iPSC-derived hepatocyte-, endothelial-, and MSC-like cells, dental pulp- derived-MSCs, and human aortic endothelial cells	Five different cell types were mixed to generate four organoid groups.	The greatest difference between four groups is the expression of phase I and phase II enzymes.		Both iPSC-derived non-parenchymal cells and adult non-parenchymal cells improved hepatic functions by mediating Wnt and TGF-β pathways. No comparison with primary hepatocytes was shown.	Goulart et al., 2019
Biomaterials and bioengineering	PSC-derived hepatocyte-like cells, HUVECs, and UC-MSCs	PSC-derived hepatic endoderm, HUVECs, and MSCs were mixed with liver decellularized matrix-derived microparticles to form LOs	Microparticles improved the maturation and metabolic capacity of PSC-derived hepatocytes.	Transplantation	No comparison with primary hepatocytes was shown.	Zahmatkesh et al., 2021
	iPSC-derived hepatocytes and iPSC-derived endothelial cells	Hepatocytes and endothelial cells were encapsulated in separate domains of fibers containing chitin, alginate, galactose, and collagen.	Integration with host vasculature *in vivo*	Liver tissue engineering	No comparison with primary hepatocytes was shown.	Du et al., 2014
	PSC-derived hepatoblasts	Hepatoblasts seeded in 500-μm diameter microwells to form uniformly sized hepatocyte-like cell (HLC) spheroids	HLC spheroids had sensitivity to various hepatotoxicants	Drug hepatotoxicity	Eight of the fifteen compounds showed higher cytotoxic activity to HLC spheroids when compared with primary hepatocytes	Lee et al., 2021

#### Heterogeneity

Heterogeneity refers to cellular, morphological, and functional non-uniformity of PSC-LOs. Heterogeneity during multi-step stem cell differentiation is a common problem and becomes even more problematic at the maturation stage of PSC differentiation into LOs. In disease modeling and drug testing, LO heterogeneity can decrease reproducibility of screens and tests. To overcome this issue, researchers have fabricated microwells to control spheroid size and uniformity (Takebe et al., 2017). Silicone microwells were recently used to generate uniform hepatocyte spheroids from human PSCs (Lee et al., 2021). These spheroids were more sensitive to liver toxins than 2D hepatocytes in image-based testing. Another study using agarose microplates also generated uniformly sized liver spheres containing human PSC-derived hepatic progenitors, hepatic stellate cells, and endothelial cells (Lucendo-Villarin et al., 2020). By utilizing automated platform, the researchers showed reduced variation and increased throughput. 3D bioprinting is a technology that uses thin layers of the cells and other components arranged on top of each other to form a complex biological structure. Because of the tools for the precise control of the process of tissue generation, and, therefore, the ability to create complex tissue patterns, 3D bioprinting has become a promising technology for transplantation, fundamental and applicable research. The most known bioprinting methods are laser pulses, extrusion, and inkjet (Kryou et al., 2019). One of the issues of 3D bioprinting for today is promoting the vascularization of a printed organ because organoids often fail to recapitulate the functionality of primary hepatocytes in the absence of vascularization. This can be solved by integrating microfluidics into bioprinting. Another issue is the stress to cells generated by 3D bioprinting tools (Faulkner-Jones et al., 2015).

#### Spatial Structure and Tissue Complexity

The majority of studies have focused on the generation of hepatocyte-like cells. However, the liver consists of multiple cell types, and for the better replication of liver structure, many recent projects have developed protocols that can produce LOs consisting of different liver cell types. A pioneer study generated 3D human liver buds by combining human iPSC-derived hepatic endoderm cells, mesenchymal stem cells, and HUVECs (Takebe et al., 2013). The hepatic functions of the generated liver buds were assessed both *in vitro* and after transplantation in mice with a liver failure model. Interestingly, interactions between multiple germ layer derivatives not only increased organoid complexity but also improved functionality. *In vivo* transplantation showed a spatial pattern expressed by these liver buds and reversed drug-induced lethal liver failure (Takebe et al., 2013, 2017). However, the organoid structure did not fully recapitulate the spatial organization of the liver, and the spatial structure also varies among organoids. Other studies also demonstrated that the combination of hepatocytes with non-parenchymal cells provided signaling interactions between cells that positively affected hepatic functions (Du et al., 2014).

Recently, Ramli et al. (2020) embedded single posterior foregut cells derived from human PSCs in Matrigel to form hepatic endoderm spheroids. Further treatment with BMP4, BMP7, and FGF7 resulted in the formation of hepatoblasts spheroids. Then, the dissociated hepatoblast spheroids were seeded into 96-well plates to form hepatic organoids consisting of hepatocytes and biliary cells with bile canaliculi. These two-cell type LOs were used in the study of nonalcoholic steatohepatitis (NASH) (Ganesh et al., 2019). Ouchi et al. (2019) took a different approach to generate multicellular LOs for the study of steatohepatitis. They first established foregut organoids from PSCs, which consisted of both endoderm and mesoderm derivatives. The foregut organoids were further differentiated into LOs composed of hepatocytes, stellate cells, and Kupffer cells. These LOs exhibited the feature of steatohepatitis when stimulated by free fatty acids. Diseased PSC-LOs were shown to be suitable for studying individualized therapy of human liver fibrosis.

Metabolic zonation is a unique feature of the liver. Liver zonation is regulated by oxygen gradient (Kietzmann, 2017), Wnt/β-catenin signaling (Planas-Paz et al., 2016), and other pathways. Liver zonation makes liver structure highly complex and spatially heterogenous and also makes *in vitro* LO generation more challenging. To our knowledge, the current technology has not been able to generate PSC-LOs mimicking a specific liver metabolic zone. This could be a future research direction to obtain LOs with desired metabolic functions for certain studies. For example, NASH affects lipid zonation and causes cell damage in the pericentral region (Hall et al., 2017). Thus, PSC-LOs representing the pericentral region would be useful in modeling NASH. Oxygen and lipid gradient created by a microfluidic chip was used to study the progress of NASH in rat primary hepatocytes (Bulutoglu et al., 2019). This microfluidic device could be integrated with human PSC-LOs consisting multiple cell types to study intercellular crosstalk during the development of NASH. Ahn et al. (2019) developed a channel system in which HepaRG cells were grown under a concentration gradient of CHIR99021, an inducer of Wnt/β-catenin pathway. This gradient created hepatic zonal environment that made HepaRG cells respond to toxic drugs differently, cells in the zone-3 region showing sensitivity to hepatotoxic drugs. It is known that ECM composition exhibits zone-dependent distribution. By adjusting ECM proportion on silk scaffold, researchers have created a hepatic zonation model that was tested with rat hepatocytes (Janani and Mandal, 2021). These are interesting models worth exploiting with human PSC-LOs to test zonal toxicity of drugs.

#### Challenges in Modeling Infectious Diseases

Due to species-dependent features of many human hepatotropic pathogens, human cells *in vitro* or in humanized chimeric animals are the only valid models to study pathogen infection and to develop therapies. Various models based on 2D or 3D cultured human primary hepatocytes, immortalized hepatic cells, and human PSC-derived hepatocytes either alone or combined with non-parenchymal cells have been established (Gural et al., 2018; Arez et al., 2021).

Earlies studies have shown the infection and replication of hepatitis C virus (HCV) in human PSC-derived hepatocytes, though these cells were not fully mature as evidenced by the expression of fetal liver markers (Roelandt et al., 2012; Schwartz et al., 2012). By closely examining HCV infection during differentiation, Wu et al. (2012) have found that undifferentiated human PSCs and DE cells were not permissive for HCV infection, but hepatic progenitors were readily infected with HCV. These studies present the application value of human PSC-derived hepatocytes in modeling HCV infection despite their unsatisfying maturity. Additionaly, when using iPSCs from patients with genetic diseases, this model can be used to study how genetic alteration affects HCV infection. Because 3D models can mimic the complexicity of the liver tissue, they show advantages in recapitulating some liver functions that 2D models cannot. However, to our knowledge, human PSC-LOs have not been used in the study of HCV-host interactions.

Primary adult human hepatocytes are the only host cell type for hepatitis B virus (HBV) infection *in vivo*, and thus they are the gold-standard for studying HBV-host interactions (Galle et al., 1994). An earlier study showed that HBV entered differentiated and polarized hepatocytes via basolateral membrane (Schulze et al., 2012). Sodium taurocholate cotransporting polypeptide (NTCP), mediating most of the Na^+^-dependent uptake of bile salts in the liver (Stieger, 2011) and being expressed on the basolateral membrane of highly differentiated hepatocytes, has been identified as a receptor for HBV (Yan et al., 2012). Because primary adult human hepatocytes were not able to maintain and expand in long-term culture, improved cell culture systems have been established to study HBV-host interactions *in vitro*. A micropatterned coculture of primary hepatocytes with stromal cells has shown support for HBV infection and enabled to study long-term HBV-host interactions (Shlomai et al., 2014). With this system, the authors have found variations of HBV infection among different donors. To study such variations in an isogenic background, the authors used human iPSC-derived hepatocytes and found that HBV infection occurred in fully differentiated hepatocytes, not in cells at earlier stages of differentiation. The maturation of hepatocytes in PSC-LOs requires assistance of their interactions with non-parenchymal cells (Si-Tayeb et al., 2010). Therefore, reconstruction of these interactions might be a feasible approach to promoting the modeling of infectious disease development. To better understand the life cycle of HBV and to develop effective anti-HBV drugs, Nie et al. (2018) have established a HBV infection model generated from human iPSC-LOs containing PSC-derived hepatocytes, mesenchymal stem cells, and HUVECs in a 3D microwell system. This infection model could support long-term replication of HBV and at the same time exhibit phenotypic alterations in hepatic functions and ultrastructure. Human iPSC-LO may still have some characteristics different from adult hepatocytes, which limits the modeling of authentic infection.

Malaria, caused by *Plasmodium* protozoan parasites, is another infectious disease related to liver. Careful examination of *Plasmodium* infection in differentiating human PSCs has demonstrated that malaria infection started from the hepatoblst stage to hepatocytes (Ng et al., 2015). However, due to the immaturity of human PSC-derived hepatocytes, they have limited potential to test anti-malaria prodrugs that require activation by hepatocyte-specific metabolizing enzymes. Using small chemicals to improve maturation, the authors showed cell response to anti-malaria prodrug primaquine, which indicates the importance of maturity for drug tesing.

In conclusion, human PSC-LOs have not yet been widely used in modeling infectious diseases partly due to their immaturity and shortage of recognition. With the improvement of maturation, PSC-LOs are expected to show more values in the study of infectious diseases and the development of therapies.

## Conclusion

**FIGURE 1:**
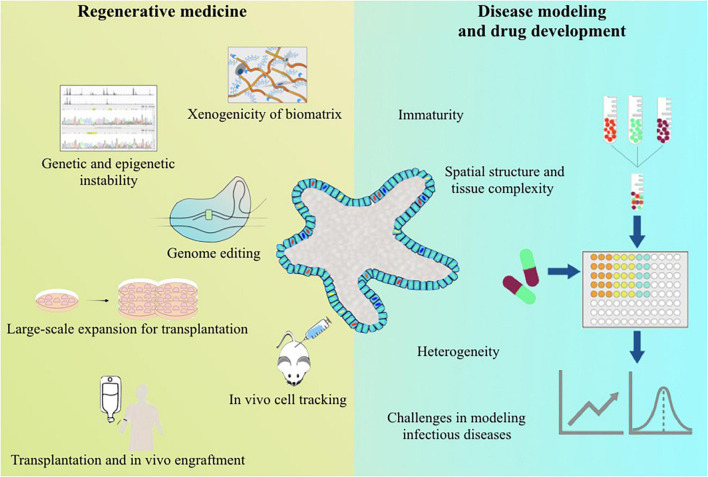
Summary of the current challenges for the applications of human pluripotent stem cell-derived liver organoids.

There is no doubt about the potential values of human PSC-LOs in regenerative medicine, disease modeling, and drug development. However, it has been very slow for human PSC-LOs moving to applications. For regenerative medicine, human PSC-LOs must be safe and effective *in vivo*, which requires the use of xeno-free materials in the generation and expansion of human PSC-LOs, reliable genome editing technique, and effective *in vivo* delivery methods (Figure 1). Meanwhile, genetic and epigenetic stability must be monitored thoroughout the generation and culture of PSC-LOs. For disease modeling and drug development, improving maturity, reducing heterogeneity, and increasing complexity are current challenges to be solved (Figure 1). By working with biomaterial scientists, bioengineers, pharmacists, and physicists, researchers can nowadays design novel 3D cell culture platforms to make human PSC-LOs suitable for intended applications. All of these will contribute to treating patients with liver diseases.

## Author Contributions

Y-RL: conceptualization, supervision, project administration, and funding acquisition. Y-RL, MC, and MB: writing—original draft preparation. Y-RL and MC: writing—review and editing. All authors have read and agreed to the published version of the manuscript.

## Conflict of Interest

The authors declare that the research was conducted in the absence of any commercial or financial relationships that could be construed as a potential conflict of interest.

## Publisher’s Note

All claims expressed in this article are solely those of the authors and do not necessarily represent those of their affiliated organizations, or those of the publisher, the editors and the reviewers. Any product that may be evaluated in this article, or claim that may be made by its manufacturer, is not guaranteed or endorsed by the publisher.
